# Validation of a rapid collagenase activity detection technique based on fluorescent quenched gelatin with synovial fluid samples

**DOI:** 10.1186/s12896-024-00869-y

**Published:** 2024-07-19

**Authors:** Miguel de la Fuente, Diego Delgado, Maider Beitia, Gabriel Barreda-Gómez, Arantxa Acera, Mikel Sanchez, Elena Vecino

**Affiliations:** 1 Department of Cell Biology and Histology, Experimental Ophthalmo-Biology Group (GOBE, www.ehu.eus/gobe), University of the Basque Country UPV/EHU, Leioa, 48940 Spain; 2 Advanced Biological Therapy Unit, Hospital Vithas Vitoria, Vitoria-Gasteiz, 01008 Spain; 3 Research and Development Division, IMG Pharma Biotech, Zamudio, Bizkaia, Spain; 4 Ikerbasque, Basque Foundation for Science, Bilbao, 48001 Spain; 5 Arthroscopic Surgery Unit, Hospital Vithas Vitoria, Vitoria-Gasteiz, 01008 Spain

**Keywords:** Collagenase activity, Gelatin, Biomarker, MMP-9, In vitro diagnosis, Point of care

## Abstract

**Background:**

Measuring collagenase activity is crucial in the field of joint health and disease management. Collagenases, enzymes responsible for collagen degradation, play a vital role in maintaining the balance between collagen synthesis and breakdown in joints. Dysregulation of collagenase activity leads to joint tissue degradation and diseases such as rheumatoid arthritis and osteoarthritis. The development of methods to measure collagenase activity is essential for diagnosis, disease severity assessment, treatment monitoring, and identification of therapeutic targets.

**Results:**

This study aimed to validate a rapid collagenase activity detection technique using synovial fluid samples. Antibody microarray analysis was initially performed to quantify the levels of matrix metalloproteinase-9 (MMP-9), a major collagenase in joints. Subsequently, the developed gelatin-based test utilizing fluorescence measurement was used to determine collagenase activity. There was a significant correlation between the presence of MMP-9 and collagenase activity. In addition, Lower Limit of Detection and Upper Limit of Detection can be preliminary estimated as 8 ng/mL and 48 ng/mL respectively.

**Conclusions:**

The developed technique offers a potential point-of-care assessment of collagenase activity, providing real-time information for clinicians and researchers. By accurately quantifying collagenase activity, healthcare professionals can optimize patient care, improve treatment outcomes, and contribute to the understanding and management of joint-related disorders. Further research and validation are necessary to establish the full potential of this rapid collagenase activity detection method in clinical practice.

## Background

Measuring collagenase activity is of utmost importance in the field of joint health and disease management. Collagenases, a group of enzymes responsible for the degradation of collagen, play a critical role in maintaining the balance between collagen synthesis and breakdown within connective tissues within the joint [1, 2]. If the homeostasis controlled by collagenases is disrupted, joint tissue degradation is provoked, causing joint diseases such as rheumatoid arthritis (RA) or osteoarthritis (OA) [3–5]. This imbalance results in the progressive erosion of articular cartilage and structural alterations.

Collagenases belong to the matrix metalloproteinase (MMP) family of proteins. The MMP family consists of various enzymes that are involved in the degradation and remodeling of the extracellular matrix (ECM), including collagen. Collagenases, including MMP-1, MMP-8, and MMP-13, primarily cleave collagen fibrils. Gelatinases, represented by MMP-2 and MMP-9, target denatured collagen (gelatin) as well as other ECM constituents. Stromelysins, such as MMP-3, MMP-10, and MMP-11, are involved in the cleavage of various ECM proteins, including proteoglycans and fibronectin. Membrane-type MMPs, exemplified by MT1-MMP, participate in the activation of proMMP-2 and proMMP-13. This orchestrated enzymatic activity is crucial for tissue development, repair, and homeostasis. However, dysregulation of MMPs is associated with pathological conditions such as cancer, arthritis, and cardiovascular diseases.

Therefore, understanding the specific targets of each MMP is essential for therapeutic strategies aimed at modulating ECM remodeling and preventing aberrant tissue changes. Among them, matrix metalloproteinase 9 (MMP-9), also known as gelatinase B, plays a significant role in various physiological and pathological processes in joints. MMP-9 is primarily involved in the breakdown of fibrillar collagens, particularly type IV and V collagens, which are components of the ECM in joint tissues. It cleaves collagen molecules at specific sites, resulting in the fragmentation and degradation of collagen fibers. During inflammatory processes, such as those of RA or OA, MMP-9 is upregulated as part of the innate immune response. It not only contributes to collagen degradation but also promotes the migration of immune cells to the site of inflammation and facilitates the release of cytokines and chemokines, amplifying the inflammatory cascade [6, 7].

In general, collagenases, including matrix metalloproteinase-1 (MMP-1), MMP-8, MMP-9, and MMP-13, play a crucial role in the molecular basis of both RA and OA [8]. These collagenases contribute to the degradation of collagen within the joint, particularly collagen type II, which is a major component of articular cartilage. Increased levels and activity of MMP-1, MMP-8, MMP-9, and MMP-13 in SF are associated with ongoing joint tissue degradation and can serve as diagnostic biomarkers for both RA and OA [9, 10].

MMP-1 primarily digests collagen type I, which is abundant in connective tissues, and III [11, 12]. MMP-8 targets collagen types I [13, 14], while MMP-9 is involved in the breakdown of collagen type IV, the main component of the basement membrane, and gelatin, a form of hydrolyzed collagen [15–17]. MMP-13 specifically degrades collagen type II, the primary component of cartilage (Table 1) [18, 19].

**Table 1:** Summary table of the most important types of MMPs in RA and OA, their names, and the types of collagens they digest

Type of MMP	Secondary name	Target
MMP-1	Collagenase-1	Collagen types I and III
MMP-8	Collagenase-2	Collagen type I
MMP-9	Gelatinase-B	Gelatin, collagen IV
MMP-13	Collagenase-3	Collagen type II

In this context, collagenases have emerged as potential therapeutic targets for the treatment of joint diseases. Modulating collagenase activity can help regulate collagen degradation, maintaining the balance between collagen turnover and tissue preservation. Researchers are exploring strategies to selectively inhibit or modulate collagenase activity, aiming to slow joint tissue degradation and potentially delay the progression of joint diseases [9, 10].

In particular, MMPs play a significant role in the homeostasis of synovial fluid (SF), particularly in the context of joint health and disease [20]. In the synovial joints, MMPs are involved in the dynamic turnover and remodeling of the ECM within the SF, a specialized lubricating fluid that surrounds and nourishes the joints [20]. These enzymes contribute to the degradation of collagen, gelatin, and proteoglycans within the SF, influencing the viscosity and composition of this fluid. They are produced by synovial cells and chondrocytes, degrade various ECM molecules (Table 1) maintaining the fluidity and functionality of the synovial fluid, ensuring proper joint lubrication and shock absorption [21–25]. MMPs are also influenced by Calcium, Ca2 + ions are crucial for the catalytic domains of MMPs, playing a vital role in the proper folding of the molecule necessary for substrate recognition and catalysis [26]. Furthermore, this element influences the activity of MMPs through various mechanisms, including binding to calmodulin, modulating integrin-mediated signaling, and participating in intracellular cascades like PKC activation [27]. It also impacts MMP gene expression by regulating transcription factors such as NFAT and AP-1 [28]. In addition, MMPs are tightly regulated by tissue inhibitors of metalloproteinases (TIMPs), balancing ECM synthesis and degradation. This balance facilitates the removal of damaged matrix components and supports the synthesis of new ones, thereby contributing to joint health and the prevention of diseases such as osteoarthritis [29, 30]. Under normal physiological conditions, MMPs help maintain the delicate balance between ECM synthesis and degradation, ensuring proper joint function. However, in conditions such as RA and OA, there is often an imbalance in MMP activity, leading to excessive degradation of the ECM in the SF. This dysregulation contributes to joint damage, inflammation, and the progression of joint diseases. Understanding the intricate interplay between MMPs and SF dynamics is crucial [30, 31].

Therefore, measuring collagenase activity plays a crucial role in diagnosing joint diseases, assessing disease severity, monitoring treatment response, and identifying therapeutic targets [32–34]. These measurements provide valuable information for clinicians, researchers, and drug developers, ultimately contributing to the understanding and management of joint-related disorders [32–34]. By accurately quantifying collagenase activity, health care professionals can optimize patient care, improve treatment outcomes, and strive to preserve joint health and functionality. The development of a rapid test to assess collagenase activity in SF holds significant importance in the field of joint health and disease management. Currently, the measurement of collagenase activity often involves complex laboratory techniques that require specialized equipment and time-consuming procedures. Among these, widely used tests include zymography, which visually assesses enzyme activity through gel electrophoresis, and fluorogenic assays, which quantitatively measure collagenase activity using specific substrates [35, 36]. Among these substrates, DQ-gelatin, FRET-based peptides, or fluorogenic peptides derived from collagen sequences, such as those containing MCA (7-methoxycoumarin-4-acetyl) and DNP (dinitrophenyl) groups, have been widely used in the study of collagenase activity as they release a fluorescent signal upon cleavage by the enzyme, allowing for real-time and quantitative measurement of enzyme activity [37–42]. Immunoassays, such as ELISAs, which detect collagenase antigens, also play a crucial role in providing quantitative insights [43]. These diagnostic tools are invaluable in diagnosing and monitoring conditions associated with collagen degradation, aiding clinicians in making informed decisions about patient management. However, a rapid test could revolutionize the diagnostic process by providing real-time, point-of-care (PoC) assessment of collagenase activity, offering numerous advantages and potential applications.

This work aimed to validate a developed rapid collagenase activity detection technique. This test is based on immobilizing fluorescent quenched gelatin (Gel-FITC) at the bottom of the wells of a microplate. When incubated with synovial fluid, the collagenases in the fluid digest the gelatin, liberating the fluorophore and increasing the fluorescent signal, which, when measured together with a calibration curve, allows the quantification of the collagenase activity of the sample normalized by the presence of the enzyme. We validated this test by comparing the collagenase activity of 22 samples with the presence of MMP-9 previously calculated with antibody microarrays (AbMAs).

The novelty of this research lies in the innovative utilization of fluorescent quenched gelatin as a substrate for collagenase detection. By immobilizing this gelatin at the bottom of microplate wells, a platform that allows for efficient and rapid assessment of collagenase activity has been created. The principle behind this technique, wherein collagenases present in synovial fluid digest the gelatin substrate, liberating a fluorophore and resulting in an increase in fluorescent signal, showcases a useful integration of molecular biology and spectroscopic methods, which offers to researchers and clinicians a valuable tool for studying enzyme kinetics and diagnosing conditions associated with collagen degradation.

The novel Gel-FITC degradation assay, which takes about 30 min, is significantly faster and requires fewer resources compared to traditional methods like zymography, fluorogenic assays, and ELISAs, which take several hours to a full day. This makes the Gel-FITC test an efficient and accessible option for real-time, point-of-care collagenase activity assessment. We selected Gel-FITC for its accessibility, ease of manufacturing, affordability, and stability, making it a practical choice for widespread use in real-time collagenase activity assessment, especially in resource-limited settings. Its simplicity and low cost align with the need for efficient diagnostic tools that can be readily deployed in various healthcare settings.

While the utilization of fluorescent quenched gelatin for collagenase detection presents promising advantages, it is also crucial to acknowledge its potential drawbacks and limitations. It should be noted that the technique’s sensitivity may vary depending on several factors, including the concentration of collagenases in the sample and the efficiency of the gelatin substrate immobilization. In addition, there is a potential batch-to-batch variation in the gelatin substrate, which might affect assay performance and result interpretation. In order to solve all this disadvantages, when verifying, validating and upscaling the manufacturing, effective quality control measures and standardized protocols would be necessary to ensure assay consistency. Furthermore, the need for specialized equipment, such as microplate readers capable of detecting fluorescence, could limit the accessibility of the technique, particularly in resource-limited settings or smaller research laboratories.

Despite these drawbacks, the innovative nature of the technique and its potential applications in enzyme kinetics and disease diagnosis warrant further investigation and optimization to maximize its utility and address existing limitations. Continued refinement and validation efforts will be essential to enhance the reliability and robustness of this collagenase detection method.

## Methods

### Synovial fluid samples

We designed a validation study in which 22 samples were analyzed. SF samples were obtained from patients who presented with joint effusion due to degenerative knee pathologies. This effusion is extracted in routine medical practice prior to the administration of intra-articular treatments. The samples were aliquoted in 0.5 mL Eppendorf tubes (#40420050, Hamburg, Germany) and stored at − 80 °C until analysis. The collection and processing of samples was carried out by medically qualified personnel after approval from the institutional review board and in strict accordance with the tenets of the Helsinki Declaration regarding research in humans. The corresponding ethical approval was obtained (Protocol No. EPA2015046) from the Ethics Committee of the Basque Country (September 2015). Informed consent was obtained from all the participants after the nature and possible consequences of the study were explained to them.

### Antibody microarray analysis of the samples

**Fig. 1:**
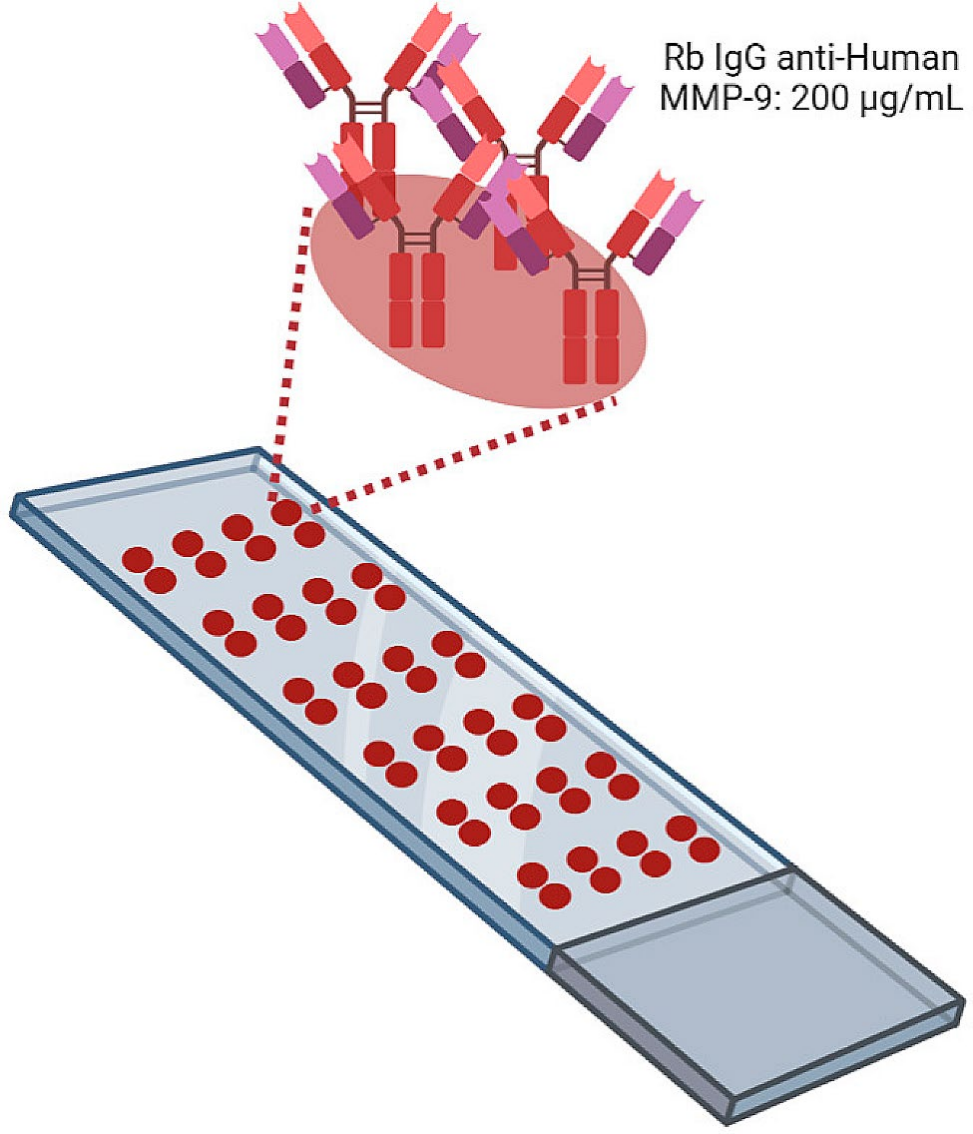
Schematic representation of a glass slide with printed AbMAs. Twenty-four AbMAs with two spots of rabbit IgG anti-human MMP-9 at 200 μg/mL in 0.05% SIVG were immobilized onto treated slides. Image created with BioRender.com

Based on our previous research with AbMAs [44–49], we analyzed the presence of the MMP-9 biomarker in the SF samples. The use of AbMAs was first validated by comparing MMP-9 biomarker quantification using an AbMA and ELISA [47]. The microarrays were fabricated on glass 76 × 26 mm microscope slides with 45° frosted ends (#1053057, LineaLAB, Badalona, Spain) preactivated with acid treatment involving different washing steps to make the surface hydrophobic (EP2048534A4, IMG Pharma Biotech S.L., Zamudio, Spain). Twenty-four AbMAs were printed onto each slide in a four-column, six-row format (Fig. 1). Each AbMA had two replicate spots of rabbit IgG anti-human MMP-9 (#10327-R043, Sino Biological, Beijing, China) immobilized at 200 μg/mL onto SIVG printing solution at 0.05% (IMG Pharma Biotech S.L., Zamudio, Spain). One drop of 30 nL was printed for each spot using a noncontact microarrayer Nano_plotter (NP 2.1., GeSiM mbH, Radeberg, Germany). The AbMAs were printed on each slide under controlled humidity (60%) at room temperature (RT) and stored at − 20 °C until use. Four slides of 24 AbMAs were immobilized on each batch. Four batch printings were carried out.

**Fig. 2:**
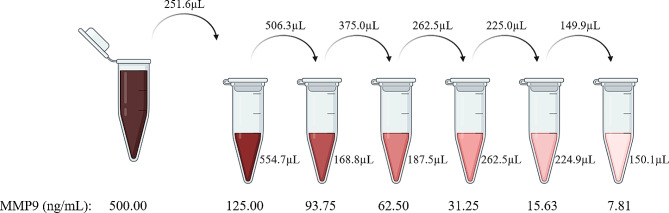
Schematic diagram of standard curve generation. Serial dilutions are made starting from a stock aliquot of 500 ng/mL of MMP-9. The volumes marked over the arrow are taken from the previous dilution and pipetted onto the previously prepared volumes marked on the bottom of the eppendorf. The final MMP-9 concentrations are indicated at the bottom of the figure

The immunodetection protocol involved first thawing and drying the slides for 30 min at RT in a drying chamber, after which they were then washed three times for 5 min with phosphate-buffered saline containing 0.01% Tween-20 (0.01% PBS-T) with agitation. The AbMAs were left in blocking solution (2.5% milk powder in 0.01% PBS-T) for 10 min at RT and then washed with distilled water. The AbMAs were then incubated overnight at 4 °C in a humid chamber with the samples diluted 1:10 in 0.5% PBS-T and 0.01% sodium dodecyl sulfate (SDS: #436143, Sigma, St. Louis, MA, USA). Alternatively, the slides were probed with the biomarker standard (MMP-9, #10327-HNAH, Sino Biological, Beijing, China) at the desired concentrations to establish calibration curves (Fig. 2).

A final volume of 20 μL was used for each AbMA. After probing, the slides were washed twice with 0.5% PBS-T and once with 0.01% PBS-T for 10 min each with agitation. The AbMAs were probed for 1 h at RT in a humid chamber with primary rabbit IgG antisera against human MMP-9 (10 μg/mL, diluted in blocking solution). After incubation, the slides were washed once with 0.5% PBS-T and twice with 0.01% PBS-T for 5 min each with agitation. The slides were incubated for 1 h at RT in a humid chamber with an Alexa Fluor 647-conjugated secondary goat anti-rabbit IgG antibody (#ab150079: Abcam, Cambridge, UK) diluted 5 μg/mL in blocking solution. Subsequently, the slides were washed with agitation for 5 min each with 0.5% PBS-T, twice with 0.01% PBS-T, once with PBS, and once with distilled water. The slides were then dried, and the fluorescent signal intensity of the spots was measured at 633 nm in an Agilent G2565BA Microarray Scanner (Agilent Technologies, Santa Clara, CA, USA). The protein concentration was determined based on the standard curve intensities. The protein concentration was determined based on the established calibration curves and using ImageLab software (Bio-Rad, Hercules, CA, USA).

### Collagenase activity of the samples

To develop a method to measure collagenase activity in human samples, gelatin-fluorescein (Gel-FITC) conjugate (#M1303, BioVision, Milpitas, CA, USA) was chosen as a substrate for the detection of gelatinases/collagenases and other gelatin-degrading enzymes. This gelatin contains the fluorophore FITC, which is quenched until the gelatin is digested when the fluorophore is released. We used P386 well microplates from Greiner (#781289, Greiner, Madrid, Spain) for the immobilization of the gelatin following the protocol detailed below.

**Fig. 3:**
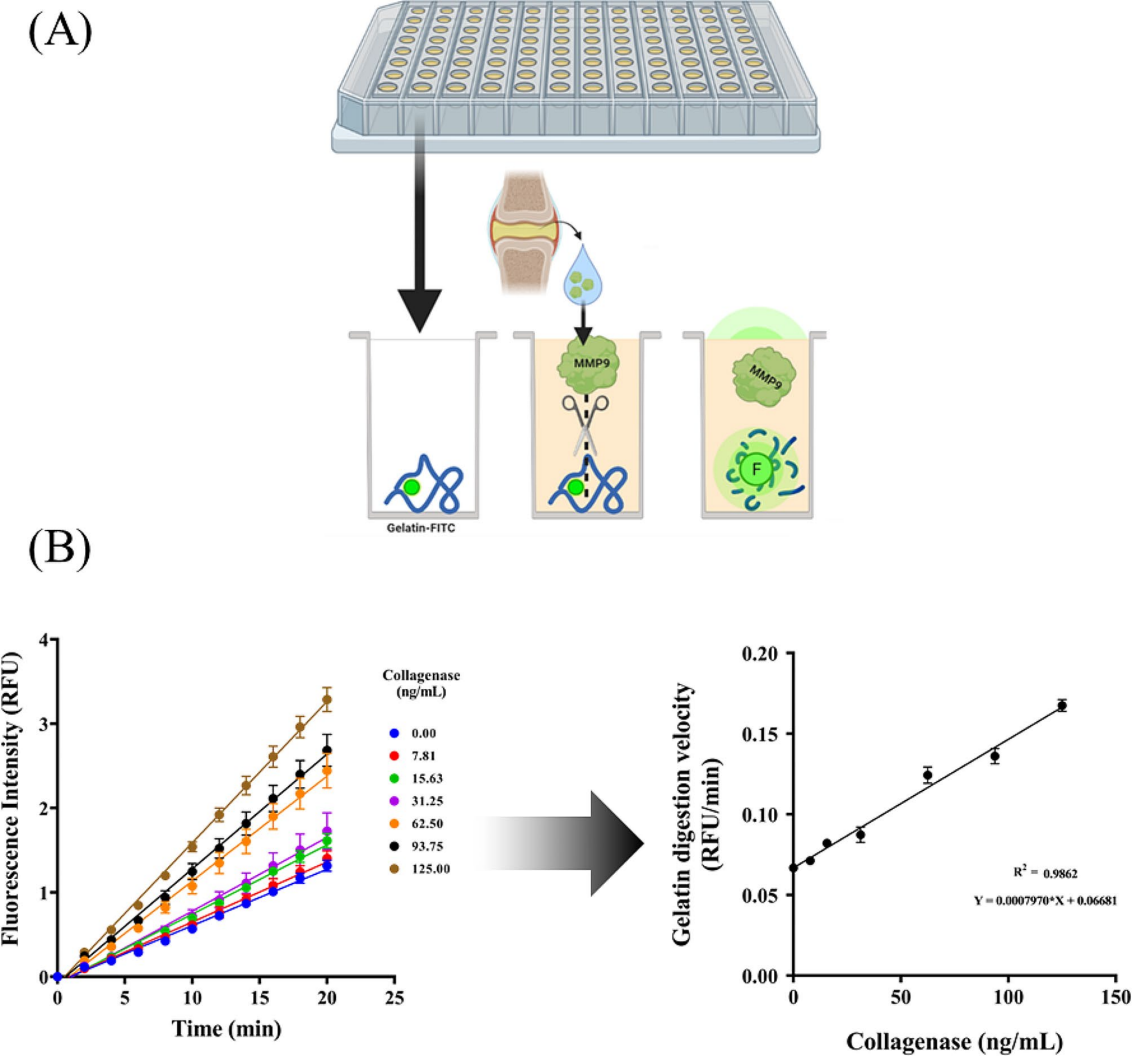
A brief summary of the protocol for the detection of the collagenase activity of the samples using quenched Gel-FITC. (**A**) Gel-FITC was added to the bottom of the wells containing P386. The samples were then loaded into each of these wells, whereby collagenases such as MMP-9 digest the gelatin, releasing the fluorophore and increasing the fluorescent signal. (**B**) The increase in the fluorescence signal over time for each collagenase standard concentration allowed the determination of the relationship between the gelatin digestion rate (RFU/min) and the presence of collagenase (ng/mL)

First, Gel-FITC was dissolved in 200 μL of distilled water, resulting in a 5 mg/mL solution, which was kept in a rotary mixer for 30 min. Then, the solution was frozen at -20 °C in 25 aliquots of 5 μL. Before use, the 5 mg/mL Gel-FITC solution was thawed at RT and diluted to a final concentration of 19 μg/mL in 0.1 M PB, pH 7.6. The solution was mixed in a rotary mixer protected from light for 30 min. Next, 50 μL of the solution was pipetted into each of the wells of the P386 microplate, and the plate was dried overnight in an oven at 40 °C for the immobilization of the Gel-FITC. Before incubation, 100 μL of enzyme incubation buffer (50 mM Tris HCl, 150 mM NaCl, 5 mM CaCl2, and 0.01% Tween, pH 7.6) was pipetted into each well of the plate for washing, the plate was incubated for 5 min in an orbital shaker, and the plate was inverted to empty the wells until the incubation buffer was removed. Finally, the samples were incubated 1:1 in enzyme incubation buffer at a final volume of 50 μL per well. Alternatively, the wells were probed with the collagenase standard (MMP-9, #10327-HNAH, Sino Biological, Beijing, China) at the desired concentrations in incubation buffer to establish calibration curves (Fig. 2). The fluorescent signal was read at 485/540 nm at 37 °C for 20 min with lectures every 2 min using a Thermo Scientific Fluoroskan Ascent FL (#1506450, Thermo Fisher Scientific, Waltham, MA, USA). A schematic representation of how this test was performed is presented in Fig. 3.

### Statistical analysis

Simple bivariate correlation was calculated when comparing Gel-FITC digestion measurements and AbMA quantifications, setting the significance at α = 0.05 using a two-tailed test for validating the reliability of the test. The data were processed with GraphPad Prism 9.2.0 (GraphPad Software, San Diego, CA, USA). Statistical analyses were carried out with SPSS 23.0 software (IBM, Armonk, NY, USA).

## Results

### Analysis of the MPP-9 concentration in synovial fluid samples by antibody microarrays

**Fig. 4:**
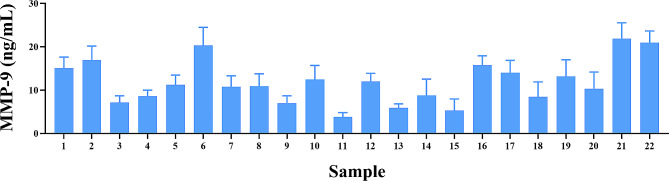
Column chart plotting the concentration of MMP-9 (ng/mL) calculated with AbMAs for each of the SF samples

The biomarker MMP-9 was analyzed in 22 synovial liquid samples using AbMAs. The concentration of each biomarker was assessed (Fig. 4), and the mean, median, maximum and minimum values were calculated (Table 2). The MMP-9 concentration was determined with AbMAs according to specific protocols. The mean concentration of MMP-9 calculated was 12.42 ng/mL, with a standard error (SEM) of 1.08 ng/mL. The median value was 11.08 ng/mL. The maximum concentration observed in the dataset was 21.89 ng/mL, and the minimum concentration was 3.78 ng/mL.

**Table 2:** The means, medians, maximum values, and minimum values of the presence of MMP-9 quantified by AbMAs in the 22 SF samples

	MMP-9 presence (ng/mL)
Mean SEM	12.42 1.08
Median	11.08
Max. value	21.89
Min. value	3.78

### Determination of collagenase activity in synovial fluid samples

**Fig. 5:**
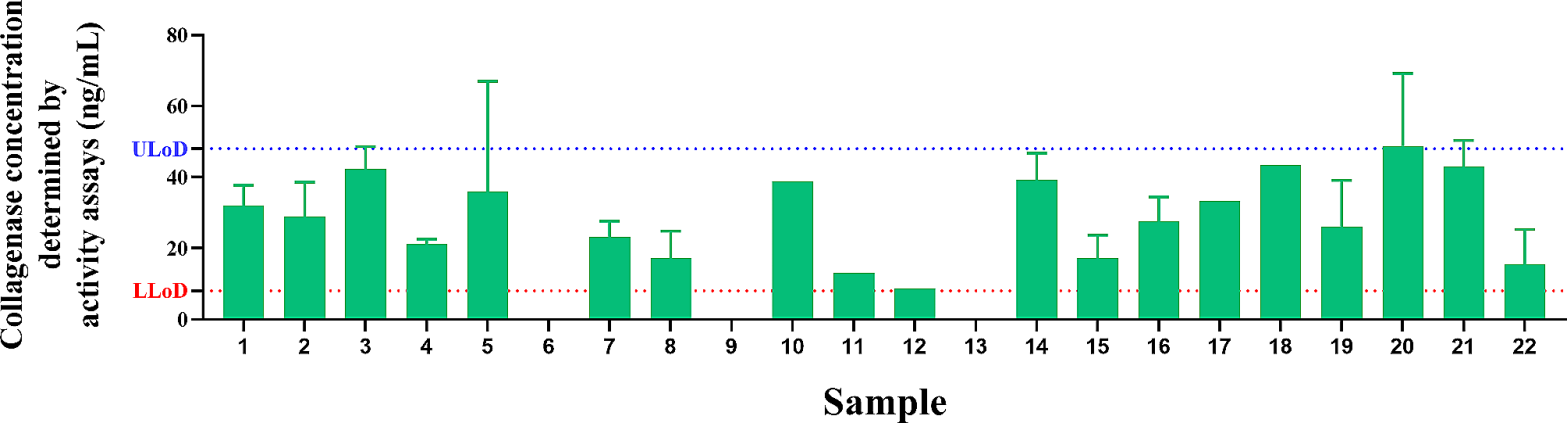
Column chart plotting the activity of collagenases normalized by enzyme presence (ng/mL) quantified by Gel-FITC digestion in the 22 SF samples under study. Samples 6, 9 and 13 showed no collagenase activity signal. LLoD can be stablished at 8 ng/mL and ULoD can be stablished at 48 ng/mL

The collagenase activity of 22 synovial liquid samples was calculated by Gel-FITC digestion. The activity of collagenases normalized by enzyme presence was assessed (Fig. 5), and the mean, median, maximum and minimum values were calculated (Table 3). Collagenase presence was calculated by Gel-FITC digestion, obtaining a mean value of 26.45 ng/mL, with a standard SEM of 3.26 ng/mL. The median value was 26.85 ng/mL. The maximum concentration observed in the dataset was 48.75 ng/mL, and the minimum concentration was 0.00 ng/mL. In addition, Lower Limit of Detection (LLoD) can be preliminarily estimated as 8 ng/mL as is the lowest collagenase concentration detected when analyzing synovial liquid clinical samples with the developed activity assay. In the same way, Upper Limit of Detection can be preliminarily estimated as 48 ng/mL (Fig. 5).

**Table 3:** The mean, median, maximum value, and minimum value of the activity of collagenases normalized by enzyme presence (ng/mL) quantified by Gel-FITC digestion in the 22 SF samples

	Collagenase concentration determined by activity assays (ng/mL)
Mean	26.45
SEM	3.26
Median	26.85
Max. value	48.75
Min. value	0.00

### Comparison between MMP-9 concentration and collagenase activity

**Fig. 6:**
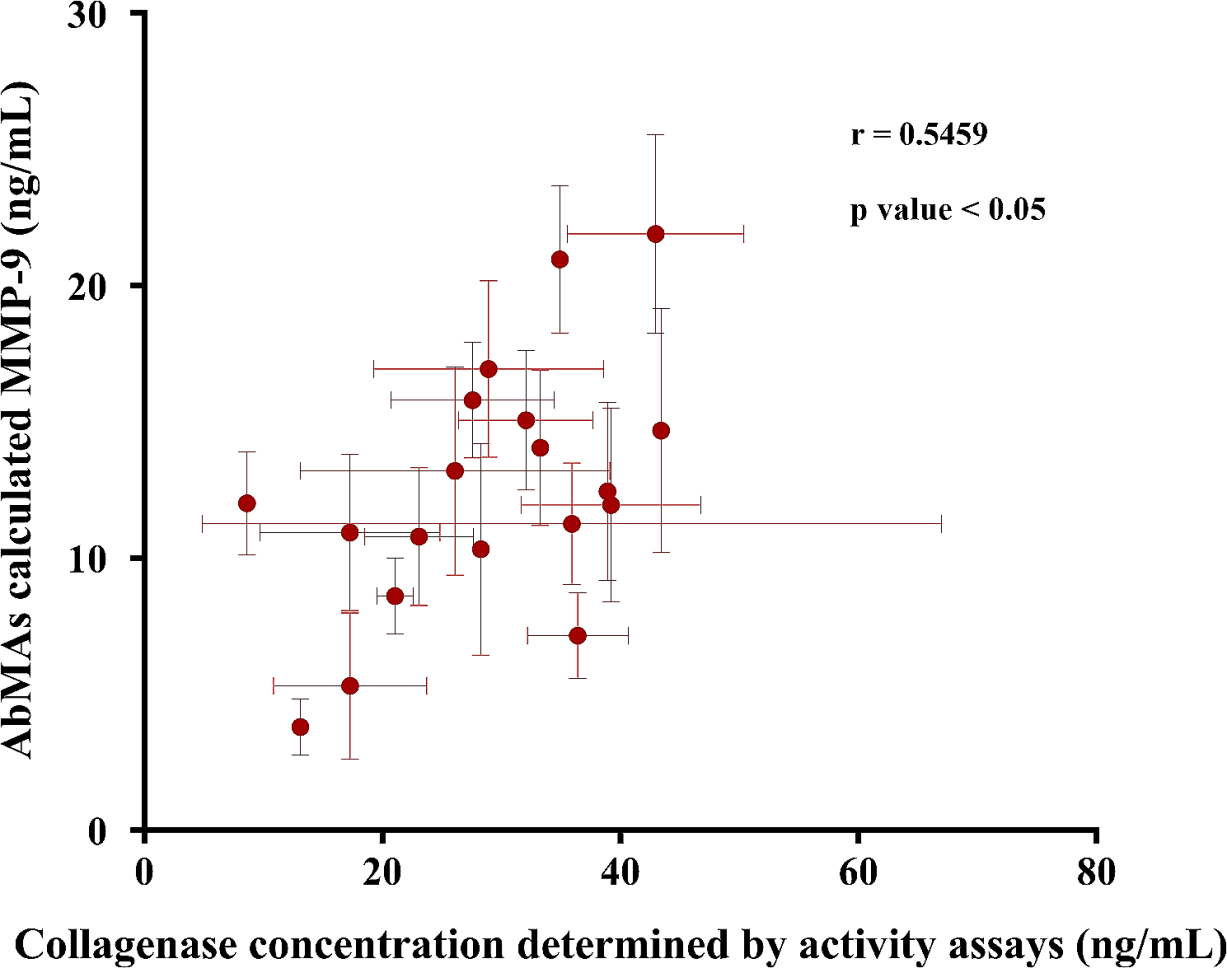
Correlation between the concentration of collagenases (ng/mL) calculated by Gel-FITC digestion using the normalized collagenase activity using an MMP-9 reference and the concentration of MMP-9 calculated with AbMAs (ng/mL)

To assess the reliability of this technique, the normalized collagenase activity values obtained were compared with the concentrations of MMP-9 collagenase determined by AbMAs in the same samples. When comparing both values, similar results were obtained (Fig. 6). Additionally, a simple bivariate correlation was calculated, setting the significance at α = 0.05 using a two-tailed test. A Pearson correlation coefficient (r) of 0.5459 was obtained with a significance of (*), and the *p* value was 0.0156 (*p* value < 0.05).

## Discussion

We developed a rapid test for immobilizing Gel-FITC at the bottom of the wells of a microplate and established a detection protocol for the quantification of collagenase activity in SF. The purpose of this test is the rapid quantification of collagenase activity as an instrument for evaluating joint diseases.

As introduced in the Background section, Gel-FITC is highly suitable due to its ease of accessibility, manufacturability, affordability, and stability. It has been used successfully in different works [50, 51]. Other substrates that can be used instead include fluorescently labeled peptides or proteins [37–42]. However, these alternatives might not have been widely adopted due to factors such as higher costs, limited availability, or lesser stability compared to Gel-FITC. Additionally, Gel-FITC’s simplicity and effectiveness make it a preferred choice for many applications.

Our results validated the developed test by comparing the normalized collagenase activity calculated by Gel-FITC digestion with the MMP-9 concentration calculated by using AbMAs. For this purpose, 22 synovial liquid samples were collected and processed using both techniques. After quantification, the observed ranges of enzyme expression and collagenase activity obtained with AbMAs and Gel-FITC, respectively, were within the ranges described in the literature, which are between 0 and 30 ng/mL [20, 50, 51]. It should be noted that the normalized collagenase activity values were greater than the MMP-9 values quantified by AbMAs. This is because not only MMP-9 but also multiple MMPs have collagenolytic activity. Other specific collagenases within the MMP family are known as MMP-1, MMP-8, and MMP-13. These collagenases, which are present in SF, are characterized by their ability to cleave and degrade fibrillar collagens, particularly type I, II, and III collagens, which are abundant in connective tissues, including those found in joints [6].

To validate the Gel-FITC degradation test, a correlation between the collagenase activity calculated by Gel-FITC digestion and the presence of MMP-9 calculated with AbMAs was performed. A statistically significant correlation in the obtained values was observed, indicating that the developed test is a useful and reliable tool for quantifying collagenase activity in human synovial liquid samples.

Collagenase activity measurements can aid in the diagnosis of joint diseases, particularly those characterized by collagen degradation, such as RA and OA. Elevated collagenase activity in the SF may indicate an imbalance in collagen turnover and degradation, indicating ongoing tissue destruction and inflammation [52–54]. Moreover, measuring collagenase activity in SF can also help differentiate between different types of arthritis. RA, for instance, is characterized by excessive synovial inflammation and upregulation of collagenase activity [55]. On the other hand, OA, a degenerative joint disease, typically exhibits lower levels of collagenase activity [34]. These distinctions can assist in accurately diagnosing and classifying specific arthritic conditions, leading to more targeted and effective therapeutic approaches. In addition, measuring collagenase activity provides insights into the extent and severity of collagen degradation within the joint [56]. Quantifying collagenase activity levels can help gauge the magnitude of tissue destruction, allowing healthcare professionals to assess disease severity and monitor disease progression over time. Higher levels of collagenase activity may indicate a more aggressive disease course and predict a poorer prognosis [57, 58]. This information can guide treatment decisions and provide prognostic indicators for patients, assisting in determining the appropriate course of action and potential interventions. Furthermore, by periodically measuring collagenase activity, healthcare providers can evaluate the effectiveness of treatments aimed at reducing joint inflammation and preventing collagen degradation. Tracking changes in collagenase activity over time provides valuable feedback on the impact of interventions, allowing for treatment adjustments and optimization of patient care [59].

With these types of collagenase activity tests, researchers can investigate the impact of various factors, such as inflammation or oxidative stress, on collagenase activity levels, providing insights into the underlying pathophysiology of joint diseases [60–62]. Moreover, these findings can guide the development of novel therapeutic interventions targeting collagenase or its regulatory mechanisms, potentially leading to the discovery of more effective treatments [63].

One of the strengths of the test developed is its speed and simplicity, which allows it to position itself as a valuable PoC. Detecting elevated collagenase activity levels in SF at an early stage may indicate ongoing joint tissue degradation before clinical symptoms become apparent. This early intervention can lead to more effective treatment strategies, helping to prevent further joint damage and improve long-term prognosis [64–66].

However, further research and validation are necessary to establish the full potential of this rapid collagenase activity detection method in clinical practice. To date, collagenase activity measurements should be interpreted in conjunction with other clinical parameters and imaging studies to form a comprehensive diagnostic picture. Combining collagenase activity analysis with imaging techniques such as magnetic resonance imaging (MRI) or ultrasound can provide a more complete assessment of joint health, aiding in accurate diagnosis and treatment planning [67–69].

With the aim of summarizing the main advantages of the developed Gel-FITC collagenase activity assay in comparison to current techniques with the same intended use, the following table (Table 4) has been constructed.

**Table 4:** Comparison between developed Gel-FITC collagenase activity assay and current techniques with the same intended use

Technique	Description	Advantages	Disadvantages
Developed Gel-FITC collagenase activity assay	Rapid collagenase activity detection technique based on immobilizing fluorescent quenched gelatin at the bottom of the wells of a microplate. When incubated with collagenases, they digest the gelatin, liberating the fluorophore, which can be measured, allowing the quantification of the collagenase activity. Its advantages make it a very good alternative to be used in clinic.	- User-friendly, easy, and fast assay. No need of expertise.	- Specific instrumentation (fluorescent lector) is needed.
		- Quantitative results achievable with appropriate calibration standards.	- It is not yet a well-established method with commercial availability.
		- Sensitive detection of enzymatic activity based on changes in fluorescence intensity.	- No yet validated with various types of samples.
		- Real-time monitoring of enzyme activity.	- Difficult to provide information about the identity of the analyzed collagenases.
Enzyme-Linked Immunosorbent Assay (ELISA) [70, 71]	ELISA involves the immobilization of a substrate specific to collagenase on a solid surface, followed by detection using an enzyme-linked antibody.	- Well-established method with commercial availability.	- Time-consuming procedure requiring several hours to complete.
		- High specificity for collagenase detection.	- Limited dynamic range, challenging for samples with low enzyme activity.
		- Compatible with various sample types and formats.	- Reliance on antibodies and substrates may introduce batch-to-batch variability and increase assay costs.
		- Quantitative results achievable with appropriate calibration standards.	- Sensitivity may vary depending on antibody affinity and substrate efficiency.
Western Blotting [72, 73]	Western blotting involves separating proteins from a sample by gel electrophoresis, followed by transfer to a membrane and detection using antibodies specific to collagenase proteins.	- Established methodology with documented protocols.	- Requires specialized equipment and expertise for gel electrophoresis and membrane transfer.
		- High specificity for detecting collagenase proteins.	- Time-consuming and labor-intensive procedure.
		- Suitable for analyzing protein expression levels and post-translational modifications.	- Sensitivity may vary depending on antibody quality and detection method.
Zymography [35, 74, 75]	Zymography involves embedding a substrate specific to collagenase in a gel matrix, followed by separation by gel electrophoresis and incubation to allow enzyme activity, visualized as clear bands.	- Specifically designed for detecting enzyme activity.	- Gel-based technique may not provide quantitative data.
		- Allows visualization of active enzyme bands directly on the gel.	- Sensitivity may vary depending on gel composition and staining method.
		- Can detect multiple enzymes simultaneously.	- Limited resolution for distinguishing closely related enzymes.
Fluorescence Resonance Energy Transfer (FRET) Assays [39–42]	FRET assays utilize changes in fluorescence intensity between fluorophores linked by a peptide sequence susceptible to collagenase cleavage to detect enzymatic activity in real-time.	- Sensitive detection of enzymatic activity based on changes in fluorescence intensity.	- Design and optimization of FRET substrates may be challenging.
		- Real-time monitoring of enzyme activity.	- Background fluorescence and assay conditions may affect signal-to-noise ratio.
		- Potential for high-throughput screening applications.	- Requires specialized equipment for fluorescence detection.
Mass Spectrometry [76]	Mass spectrometry is used to detect and quantify peptide fragments generated by enzymatic cleavage of substrates, providing information about the identity and quantity of collagenase activity in a sample.	- High sensitivity and specificity for detecting peptide fragments generated by enzymatic cleavage.	- Expensive instrumentation and expertise required.
		- Can provide information about the identity and quantity of collagenase activity in a sample.	- Sample preparation may be complex and time-consuming.
		- Suitable for identifying novel enzyme substrates.	- Interpretation of mass spectrometry data may require bioinformatics expertise.

In summary, the development of a rapid test to assess collagenase activity in SF holds immense importance in joint health. It can enable early diagnosis, aid in treatment monitoring, facilitate personalized care, identify therapeutic targets, and advance research in the field. By providing quick and reliable results, a rapid collagenase activity test has the potential to revolutionize the diagnosis and management of joint diseases, leading to improved patient outcomes and enhanced quality of life. In order to market current Gel-FITC collagenase assay, full package of characterization studies will be performed following CLSI guidelines to determine: Linearity (EP06-A), Detection Capability (EP17-A2), Precision (EP05-A3), Interfering substances (EP07-A3), Method Comparison (EP09-A3/EP12-A2), Bias (EP10-A3), and Stability (EP25-A) [77].

## Conclusion

A rapid Gel-FITC degradation test was developed for synovial liquid collagenase activity quantification in human samples. The test was validated through comparison with AbMAs. With the obtained data, we confirmed the reliability of our Gel-FITC test for the quantification of collagenase activity in human synovial liquid samples. The measurement of this parameter offers a valuable diagnostic tool for assessing joint health and detecting various arthritic conditions. By providing insights into collagen turnover and tissue destruction, these measurements can assist in early diagnosis, differentiation between different types of arthritis, and monitoring treatment response. As research continues to unveil the intricacies of collagenase activity, it holds immense potential to revolutionize the diagnosis and management of joint-related disorders, leading to improved patient outcomes and enhanced quality of life.

## Data Availability

No datasets were generated or analysed during the current study.

## References

[1] Holmbeck K, Birkedal-Hansen H. Collagenases. Encyclopedia Biol Chemistry: Second Ed. 2013;542–4. 10.1016/B978-0-12-378630-2.00008-6

[2] Abe S, Shinmei M, Nagai Y. Synovial collagenase and joint diseases: the significancy of latent collagenase with special reference to rheumatoid arthritis. J Biochem. 1973;73:1007–11. 10.1093/OXFORDJOURNALS.JBCHEM.A130154 4124619 10.1093/OXFORDJOURNALS.JBCHEM.A130154

[3] Pelletier J-P, Martel‐Pelletier J, Howell DS, Ghandur‐Mnaymneh L, Enis JE, Frederick Woessner J. Collagenase and collagenolytic activity in human osteoarthritic cartilage. Arthritis Rheum. 1983;26:63–8. 10.1002/ART.1780260110 6297508 10.1002/ART.1780260110

[4] Harris ED, Cohen GL, Krane SM. Synovial collagenase: its presence in culture from joint disease of diverse etiology. Arthritis Rheum. 1969;12:92–102. 10.1002/ART.1780120206 4305063 10.1002/ART.1780120206

[5] Vincenti MP, Brinckerhoff CE. Transcriptional regulation of collagenase (MMP-1, MMP-13) genes in arthritis: integration of complex signaling pathways for the recruitment of gene-specific transcription factors. Arthritis Res. 2002;4:157–64. 10.1186/AR401/FIGURES/2 12010565 10.1186/AR401/FIGURES/2 PMC128926

[6] Löffek S, Schilling O, Franzke CW. Biological role of matrix metalloproteinases: a critical balance. Eur Respir J. 2011;38:191–208. 10.1183/09031936.00146510 21177845 10.1183/09031936.00146510

[7] Kim KS, Choi HM, Lee YA, Choi IA, Lee SH, Hong SJ, Yang HI, Yoo MC. Expression levels and association of gelatinases MMP-2 and MMP-9 and collagenases MMP-1 and MMP-13 with VEGF in synovial fluid of patients with arthritis. Rheumatol Int. 2011;31:543–7. 10.1007/S00296-010-1592-1 20665024 10.1007/S00296-010-1592-1

[8] Yamamoto K, Wilkinson D, Bou-Gharios G. Targeting dysregulation of metalloproteinase activity in osteoarthritis. Calcif Tissue Int. 2021;109:277. 10.1007/S00223-020-00739-7 32772139 10.1007/S00223-020-00739-7 PMC8403128

[9] Karila T, Tervahartiala T, Cohen B, Sorsa T. The collagenases: are they tractable targets for preventing cartilage destruction in osteoarthritis? 10.1080/14728222. 2022;26:93–105, 10.1080/14728222.2022.2035362 10.1080/14728222.2022.2035362 35081858

[10] Hu Q, Ecker M. Overview of MMP-13 as a promising target for the treatment of osteoarthritis. Int J Mol Sci. 2021;22:1–22. 10.3390/IJMS22041742 10.3390/IJMS22041742 PMC7916132 33572320

[11] Boraschi-Diaz I, Wang J, Mort JS, Komarova SV. Collagen type I as a ligand for receptor-mediated signaling. Front Phys. 2017;5. 10.3389/FPHY.2017.00012/BIBTEX

[12] Ohuchi E, Imai K, Fujii Y, Sato H, Seiki M, Okada Y. Membrane type 1 matrix metalloproteinase digests interstitial collagens and other extracellular matrix macromolecules. J Biol Chem. 1997;272:2446–51. 10.1074/JBC.272.4.2446 8999957 10.1074/JBC.272.4.2446

[13] Lu KG, Stultz CM. Insight into the degradation of type-I collagen fibrils by MMP-8. J Mol Biol. 2013;425:1815–25. 10.1016/J.JMB.2013.02.002 23399546 10.1016/J.JMB.2013.02.002

[14] Hedenbjörk-Lager A, Hamberg K, Pääkkönen V, Tjäderhane L, Ericson D. Collagen degradation and preservation of MMP-8 activity in human dentine matrix after demineralization. Arch Oral Biol. 2016;68:66–72. 10.1016/J.ARCHORALBIO.2016.04.003 27105041 10.1016/J.ARCHORALBIO.2016.04.003

[15] Zeng ZS, Cohen AM, Guillem JG. Loss of basement membrane type IV collagen is associated with increased expression of metalloproteinases 2 and 9 (MMP-2 and MMP-9) during human colorectal tumorigenesis. Carcinogenesis. 1999;20:749–55. 10.1093/CARCIN/20.5.749 10334190 10.1093/CARCIN/20.5.749

[16] Heikinheimo K, Salo T. Expression of basement membrane type IV collagen and type IV collagenases (MMP-2 and MMP-9) in human fetal teeth. J Dent Res. 1995;74:1226–34. 10.1177/00220345950740051301 7790601 10.1177/00220345950740051301

[17] Nyante SJ, Wang T, Tan X, Ozdowski EF, Lawton TJ. Quantitative expression of MMPs 2, 9, 14, and Collagen IV in LCIS and paired normal breast tissue. Scientific Reports. 2019;9:1–9, 10.1038/s41598-019-48602-6 10.1038/s41598-019-48602-6 PMC6748975 31530842

[18] Zhang M, Zhou Q, Liang QQ, Li CG, Holz JD, Tang D, Sheu TJ, Li TF, Shi Q, Wang YJ. IGF-1 regulation of type II collagen and MMP-13 expression in rat endplate chondrocytes via distinct signaling pathways. Osteoarthritis Cartilage. 2009;17:100–6. 10.1016/J.JOCA.2008.05.007 18595745 10.1016/J.JOCA.2008.05.007

[19] Wang J, Ma J, Gu JH, Wang FY, Shang XS, Tao HR, Wang X. Regulation of type II collagen, matrix metalloproteinase-13 and cell proliferation by interleukin-1β is mediated by curcumin via inhibition of NF-ΚB signaling in rat chondrocytes. Mol Med Rep. 2017;16. 10.3892/MMR.2017.6771 10.3892/mmr.2017.6771 PMC5562050 28627596

[20] Tchetverikov I, Lohmander LS, Verzijl N, Huizinga TWJ, Tekoppele JM, Hanemaaijer R, Degroot JMMP. Protein and activity levels in synovial fluid from patients with joint injury, inflammatory arthritis, and osteoarthritis. 10.1136/ard.2004.022434 10.1136/ard.2004.022434 PMC1755474 15834054

[21] Otero M, Goldring MB. Cells of the synovium in rheumatoid arthritis. Chondrocytes. Arthritis Res Ther. 2007;9:220. 10.1186/AR2292 18001488 10.1186/AR2292 PMC2212563

[22] Önnheim K, Huang S, Strid Holmertz A, Andersson S, Lönnblom E, Jonsson C, Holmdahl R, Gjertsson I. Rheumatoid arthritis chondrocytes produce increased levels of pro-inflammatory proteins. Osteoarthr Cartil Open. 2022;4:100235. 10.1016/J.OCARTO.2022.100235 36474471 10.1016/J.OCARTO.2022.100235 PMC9718183

[23] Akkiraju H, Nohe A. Role of chondrocytes in cartilage formation, progression of osteoarthritis and cartilage regeneration. J Dev Biol. 2015;3:177. 10.3390/JDB3040177 27347486 10.3390/JDB3040177 PMC4916494

[24] Ishikawa S, Hiraizumi Y, Fujimaki E. Production of matrix metalloproteinase (MMP-2, 9) from cultured human synovial cells. Showa Univ J Med Sci. 1999;11:2–7. 10.15369/sujms1989.11.207

[25] Bondeson J, Wainwright SD, Lauder S, Amos N, Hughes CE. The role of synovial macrophages and macrophage-produced cytokines in driving aggrecanases, matrix metalloproteinases, and other destructive and inflammatory responses in osteoarthritis. Arthritis Res Ther. 2006;8:R187. 10.1186/AR2099 17177994 10.1186/AR2099 PMC1794533

[26] Meraz-Cruz N, Ortega A, Estrada-Gutierrez G, Flores A, Espejel A, Hernandez-Guerrero C, Vadillo-Ortega F. Identification of a calcium-dependent matrix metalloproteinase complex in rat chorioallantoid membranes during labour. Mol Hum Reprod. 2006;12:633–41. 10.1093/MOLEHR/GAL072 16935996 10.1093/MOLEHR/GAL072

[27] Tebar F, Lladó A, Enrich C. Role of calmodulin in the modulation of the mapk signalling pathway and the transactivation of epidermal growth factor receptor mediated by PKC. FEBS Lett. 2002;517:206–10. 10.1016/S0014-5793(02)02624-8 12062438 10.1016/S0014-5793(02)02624-8

[28] Park YJ, Yoo SA, Kim M, Kim WU. The role of calcium–Calcineurin–NFAT signaling pathway in health and autoimmune diseases. Front Immunol. 2020;11:455685. 10.3389/FIMMU.2020.00195/BIBTEX 10.3389/FIMMU.2020.00195/BIBTEX PMC7075805 32210952

[29] Plsikova Matejova J, Spakova T, Harvanova D, Lacko M, Filip V, Sepitka R, Mitro I, Rosocha J. A preliminary study of combined detection of COMP, TIMP-1, and MMP-3 in synovial fluid: potential indicators of osteoarthritis progression. Cartilage. 2021;13:S1421–30. 10.1177/1947603520946385 10.1177/1947603520946385 PMC8804792 32748631

[30] Fuchs S, Skwara A, Bloch M, Dankbar B. Differential induction and regulation of matrix metalloproteinases in osteoarthritic tissue and fluid synovial fibroblasts. Osteoarthritis Cartilage. 2004;12:409–18. 10.1016/J.JOCA.2004.02.005 15094140 10.1016/J.JOCA.2004.02.005

[31] Mukherjee A, Das B. The role of inflammatory mediators and matrix metalloproteinases (MMPs) in the progression of osteoarthritis. Biomaterials Biosystems. 2024;13:100090. 10.1016/J.BBIOSY.2024.100090 38440290 10.1016/J.BBIOSY.2024.100090 PMC10910010

[32] Harris ED, Faulkner CS, Brown FE. Collagenolytic systems in rheumatoid arthritis. Clin Orthop Relat Res. 1975;110:303–16. 10.1097/00003086-197507000-00041 10.1097/00003086-197507000-00041 168997

[33] Maeda S, Sawai T, Uzuki M, Takahashi Y, Omoto H, Seki M, Sakurai M. Determination of interstitial collagenase (MMP-1) in patients with rheumatoid arthritis. Ann Rheum Dis. 1995;54:970–5. 10.1136/ARD.54.12.970 8546529 10.1136/ARD.54.12.970 PMC1010062

[34] Clark IM, Powell LK, Ramsey S, Hazleman BL, Cawston TE. The measurement of collagenase, tissue inhibitor of metalloproteinases (Timp), and collagenase—timp complex in synovial fluids from patients with osteoarthritis and rheumatoid arthritis. Arthritis Rheum. 1993;36:372–9. 10.1002/ART.1780360313 8452582 10.1002/ART.1780360313

[35] Tajhya RB, Patel RS, Beeton C. Detection of matrix metalloproteinases by zymography. Methods Mol Biol. 2017;1579:231. 10.1007/978-1-4939-6863-3_12 28299740 10.1007/978-1-4939-6863-3_12 PMC5465868

[36] Makowski GS, Ramsby ML. Zymographic analysis of latent and activated forms of matrix metalloproteinase-2 and – 9 in synovial fluid: correlation to polymorphonuclear leukocyte infiltration and in response to infection. Clin Chim Acta. 2003;329:77–81. 10.1016/S0009-8981(03)00015-9 12589968 10.1016/S0009-8981(03)00015-9

[37] Vandooren J, Geurts N, Martens E, Steen PE van den, Jonghe S De, Herdewijn P, Opdenakker G. Gelatin degradation assay reveals MMP-9 inhibitors and function of O-glycosylated domain. World J Biol Chem. 2011;2:14. 10.4331/WJBC.V2.I1.14 21537473 10.4331/WJBC.V2.I1.14 PMC3083944

[38] Singh N, Bhattacharyya D. Collagenases in an ether extract of bacterial metabolites used as an immunostimulator induces TNF-α and IFN-γ. Int Immunopharmacol. 2014;23:211–21. 10.1016/J.INTIMP.2014.08.026 25203593 10.1016/J.INTIMP.2014.08.026

[39] Fields GB. Using fluorogenic peptide substrates to assay matrix metalloproteinases. Methods Mol Biol. 2001;151:495. 10.1385/1-59259-046-2:495 11217324 10.1385/1-59259-046-2:495 PMC4354778

[40] Tokmina-Roszyk M, Tokmina-Roszyk D, Bhowmick M, Fields GB. Development of a fluorescence resonance energy transfer assay for monitoring bacterial collagenase triple-helical peptidase activity. Anal Biochem. 2014;453. 10.1016/J.AB.2014.02.024 10.1016/j.ab.2014.02.024 PMC4260936 24608089

[41] Liu H, Liang G, Abdel-Halim ES, Zhu JJ. A sensitive and selective quantum dots-based FRET Biosensor for the detection of cancer marker type IV collagenase. Anal Methods. 2011;3:1797–801. 10.1039/C1AY05178D 10.1039/C1AY05178D

[42] Al-Abdullah IH, Bagramyan K, Bilbao S, Qi M, Kalkum M. Fluorogenic peptide substrate for quantification of bacterial enzyme activities. Sci Rep. 2017;7. 10.1038/SREP44321 10.1038/srep44321 PMC5347087 28287171

[43] Yoshioka H, Oyamada I, Usuku G. An assay of collagenase activity using enzyme-linked immunosorbent assay for mammalian collagenase. Anal Biochem. 1987;166:172–7. 10.1016/0003-2697(87)90559-8 2823639 10.1016/0003-2697(87)90559-8

[44] Acera A, Rocha G, Vecino E, Lema I, Durán JA. Inflammatory markers in the tears of patients with ocular surface disease. Ophthalmic Res. 2008;40:315–21. 10.1159/000150445 18688174 10.1159/000150445

[45] Acera A, Vecino E, Duran JA, Tear. MMP-9 levels as a marker of ocular surface inflammation in conjunctivochalasis. Invest Ophthalmol Vis Sci. 2013;54:8285–91. 10.1167/iovs.13-12235 24255042 10.1167/iovs.13-12235

[46] Acera A, Suárez T, Rodríguez-Agirretxe I, Vecino E, Durán JA. Changes in tear protein profile in patients with conjunctivochalasis. Cornea. 2011;30:42–9. 10.1097/ICO.0B013E3181DEA7D7 20861728 10.1097/ICO.0B013E3181DEA7D7

[47] de la Fuente M, Rodríguez-Agirretxe I, Vecino E, Astigarraga E, Acera A, Barreda-Gómez G. Elevation of tear MMP-9 concentration as a biomarker of inflammation in ocular pathology by antibody microarray immunodetection assays. Int J Mol Sci. 2022;23:5639. 10.3390/ijms23105639 35628448 10.3390/ijms23105639 PMC9147659

[48] Joachim SC, Hohberger B, Sanderson J, Unterlauft JD, Wareham LK, Boto De Los Bueis A, De La Fuente M, Montejano-Milner R, Del Hierro Zarzuelo A, Vecino E, et al. A pilot study of a panel of ocular inflammation biomarkers in patients with primary Sj&ouml;Gren&rsquo;s syndrome. Curr Issues Mol Biol. 2023;45:2881–2894. 10.3390/CIMB45040188 10.3390/cimb45040188 PMC10136698 37185712

[49] Manuel Benitez-Del-Castillo J, Soria J, Acera A, María Muñoz A, Rodríguez S, Suárez T. *Quantification of a Panel for Dry-Eye Protein Biomarkers in Tears: A Comparative Pilot Study Using Standard ELISA and Customized Microarrays*; 2021. PMC8116255 34012227

[50] Stojanovic SK, Stamenkovic BN, Cvetkovic JM, Zivkovic VG, Andjelkovic Apostolovic MR. Matrix metalloproteinase-9 level in synovial fluid-association with joint destruction in early rheumatoid arthritis. 2023. 10.3390/medicina59010167 10.3390/medicina59010167 PMC9863294 36676791

[51] Tchetverikov I, Ronday HK, Van El B, Kiers GH, Verzijl N, Tekoppele JM, Huizinga TWJ, Degroot J, Hanemaaijer R. MMP profile in paired serum and synovial fluid samples of patients with rheumatoid arthritis. Ann Rheum Dis. 2004;63:881–3. 10.1136/ard.2003.013243 15194590 10.1136/ard.2003.013243 PMC1755080

[52] Lindy S, Turto H, Sorsa T, Halme J, Lauhio A, Suomalainen K, Uitto VJ, Wegelius O. Increased collagenase activity in human rheumatoid meniscus. Scand J Rheumatol. 1986;15:237–42. 10.3109/03009748609092585 3026034 10.3109/03009748609092585

[53] Ahrens D, Koch AE, Pope RM, Stein-Picarella M, Niedbala MJ. Expression of matrix metalloproteinase 9 (96-Kd gelatinase B) in human rheumatoid arthritis. Arthritis Rheum. 1996;39:1576–87. 10.1002/ART.1780390919 8814070 10.1002/ART.1780390919

[54] Mahmoud RA, EL-Ansary A, El-Eishi HH, Kamal HM, El-Saeed NH. Matrix metalloproteinases MMP-3 and MMP-1 levels in sera and synovial fluids in patients with rheumatoid arthritis and osteoarthritis. Ital J Biochem. 2005. 16688934

[55] Sweeney SE, Firestein GS, Rheumatoid Arthritis. Regulation of synovial inflammation. Int J Biochem Cell Biology. 2004;36:372–8. 10.1016/S1357-2725(03)00259-0 10.1016/S1357-2725(03)00259-0 14687914

[56] Hakala M, Risteli L, Manelius J, Nieminen P, Risteli J. Increased type I collagen degradation correlates with disease severity in rheumatoid arthritis. Ann Rheum Dis. 1993;52:866–9. 10.1136/ARD.52.12.866 8311537 10.1136/ARD.52.12.866 PMC1005217

[57] Kotaniemi A, Isomaki H, Hukala M, Risteli L, Risteli J. Increased type I collagen degradation in early rheumatoid arthritis. J Rheumatol. 1994;21:1593–6. 7799334

[58] Aman S, Hakala M, Risteli L, Risteli J, Increased Type I. Collagen degradation is associated with a need for total joint replacement surgery in rheumatoid arthritis. Ann Rheum Dis. 1996;55:147. 10.1136/ARD.55.2.147-A 8712868 10.1136/ARD.55.2.147-A PMC1010112

[59] Greenwald RA. Monitoring collagen degradation in patients with arthritis. The search for suitable surrogates. Arthritis Rheum. 1996;39:1455–65. 10.1002/ART.1780390904 8814056 10.1002/ART.1780390904

[60] Fisher GJ, Quan T, Purohit T, Shao Y, Moon KC, He T, Varani J, Kang S, Voorhees JJ. Collagen fragmentation promotes oxidative stress and elevates matrix metalloproteinase-1 in fibroblasts in aged human skin. Am J Pathol. 2009;174:101. 10.2353/AJPATH.2009.080599 19116368 10.2353/AJPATH.2009.080599 PMC2631323

[61] Siwik DA, Pagano PJ, Colucci WS. Oxidative stress regulates collagen synthesis and matrix metalloproteinase activity in cardiac fibroblasts. Am J Physiol Cell Physiol. 2001;280. 10.1152/AJPCELL.2001.280.1.C53 10.1152/ajpcell.2001.280.1.C53 11121376

[62] Schock BC, Sweet DG, Ennis M, Warner JA, Young IS, Halliday HL. Oxidative stress and increased type-IV collagenase levels in bronchoalveolar lavage fluid from newborn babies. Pediatr Res. 2001;50:29–33. 10.1203/00006450-200107000-00008 10.1203/00006450-200107000-00008 11420415

[63] Alipour H, Raz A, Zakeri S, Dinparast Djadid N. Therapeutic applications of collagenase (metalloproteases): a review. Asian Pac J Trop Biomed. 2016;6:975–81. 10.1016/J.APJTB.2016.07.017 10.1016/J.APJTB.2016.07.017

[64] Chu CR, Williams AA, Coyle CH, Bowers ME. Early diagnosis to enable early treatment of pre-osteoarthritis. Arthritis Res Ther. 2012;14:212. 10.1186/AR3845 22682469 10.1186/AR3845 PMC3446496

[65] Chu CR, Millis MB, Olson SA, Osteoarthritis. From palliation to prevention: AOA critical issues. J Bone Joint Surg Am. 2014;96(1). 10.2106/JBJS.M.01209 10.2106/JBJS.M.01209 PMC4116563 25100783

[66] Kandahari AM, Yang X, Dighe AS, Pan D, Cui Q. Recognition of immune response for the early diagnosis and treatment of osteoarthritis. J Immunol Res. 2015;2015. 10.1155/2015/192415 10.1155/2015/192415 PMC4433702 26064995

[67] Jerban S, Chang EY, Du J. Magnetic resonance imaging (MRI) studies of knee joint under mechanical loading: review. Magn Reson Imaging. 2020;65:27–36. 10.1016/J.MRI.2019.09.007 31670237 10.1016/J.MRI.2019.09.007 PMC6938531

[68] Østergaard M, Boesen M. Imaging in rheumatoid arthritis: the role of magnetic resonance imaging and computed tomography. Radiol Med. 2019;124:1128–41. 10.1007/S11547-019-01014-Y 30880357 10.1007/S11547-019-01014-Y

[69] Sudoł-Szopińska I, Jans L, Teh J. Rheumatoid arthritis: what do MRI and ultrasound show. J Ultrason. 2017;17. 10.15557/JOU.2017.0001 10.15557/JoU.2017.0001 PMC5392548 28439423

[70] Bergmann U, Michaelis J, Oberhoff R, Knäuper V, Beckmann R, Tschesche H. Enzyme linked immunosorbent assays (ELISA) for the quantitative determination of human leukocyte collagenase and gelatinase. J Clin Chem Clin Biochem. 1989;27:351–60. 10.1515/CCLM.1989.27.6.351 2547014 10.1515/CCLM.1989.27.6.351

[71] Cooper TW, Bauer EA, Eisen AZ. Enzyme-linked immunosorbent assay for human skin collagenase. Coll Relat Res. 1983;3:205–15. 10.1016/S0174-173X(83)80004-1 6191911 10.1016/S0174-173X(83)80004-1

[72] Balbín M, Fueyo A, Knäuper V, Pendás AM, López JM, Jiménez MG, Murphy G, López-Otín C. Collagenase 2 (MMP-8) expression in murine tissue-remodeling processes. Analysis of its potential role in postpartum involution of the uterus. J Biol Chem. 1998;273:23959–68. 10.1074/JBC.273.37.23959 9727011 10.1074/JBC.273.37.23959

[73] Kassegne K, Hu W, Ojcius DM, Sun D, Ge Y, Zhao J, Yang XF, Li L, Yan J. Identification of collagenase as a critical virulence factor for invasiveness and transmission of pathogenic leptospira species. J Infect Dis. 2014;209:1105–15. 10.1093/INFDIS/JIT659 24277745 10.1093/INFDIS/JIT659

[74] Ricci S, D’Esposito V, Oriente F, Formisano P, Di Carlo A, Substrate-Zymography. A still worthwhile method for gelatinases analysis in biological samples. Clin Chem Lab Med. 2016;54:1281–90. 10.1515/CCLM-2015-0668 26641968 10.1515/CCLM-2015-0668

[75] Ren Z, Chen J, Khalil RA. Zymography as a research tool in the study of matrix metalloproteinase inhibitors. Methods Mol Biol. 2017;1626:79. 10.1007/978-1-4939-7111-4_8 28608202 10.1007/978-1-4939-7111-4_8 PMC5527288

[76] Clift CL, Drake RR, Mehta A, Angel PM. Multiplexed imaging mass spectrometry of the extracellular matrix using serial enzyme digests from formalin-fixed paraffin embedded tissue sections. Anal Bioanal Chem. 2021;413:2709. 10.1007/S00216-020-03047-Z 33206215 10.1007/S00216-020-03047-Z PMC8012227

[77] Clinical & Laboratory Standards Institute: CLSI Guidelines Available online: https://clsi.org/ (accessed on 12. May 2024).

